# Integrated gut microbiome and serum lipidomics reveals microbial–lipid interactions for predicting incident metabolic syndrome: a nested case–control study

**DOI:** 10.3389/fmicb.2026.1862738

**Published:** 2026-08-06

**Authors:** Peimeng Zhu, Jingfeng Chen, Hang Yan, Tiantian Li, Xinxin Gao, Ang Li, Suying Ding

**Affiliations:** 1 Health Management Center, The First Affiliated Hospital of Zhengzhou University, Zhengzhou, Henan, China; 2 Department of Epidemiology, College of Public Health, Zhengzhou University, Zhengzhou, Henan, China; 3 Gene Hospital of Henan Province, The First Affiliated Hospital of Zhengzhou University, Zhengzhou, China

**Keywords:** gut microbiota, lipidomics, machine learning, metabolic syndrome, multi-omics integration, nested case–control study

## Abstract

**Background:**

Metabolic syndrome (MetS) is a multifactorial disorder characterized by obesity, dyslipidemia, hypertension, and insulin resistance. Although gut microbiota and lipid metabolism are both known to influence MetS development, their interactions remain incompletely characterized.

**Methods:**

We conducted an exploratory nested case–control study within a prospective health examination cohort. We selected 100 participants (50 incident MetS cases and 50 matched controls) based on age, sex, and baseline MetS components. Gut microbial profiles were characterized by metagenomic sequencing, and serum lipid metabolites were measured using high-resolution mass spectrometry. Multi-omics integration was performed using correlation-based feature fusion. We constructed a support vector machine (SVM) model, optimized with recursive feature elimination (RFE) and five-fold cross-validation, to predict the incidence risk of MetS.

**Results:**

MetS participants differed from controls in gut microbial composition, metabolic pathway activities, and lipidomic profiles. Circos analysis revealed positive associations between Blautia and sphingomyelins and negative associations between Bacteroides and triglycerides. The integrated model combining microbiota and lipidomic features demonstrated strong discrimination in the training set (AUC = 0.995, 95% CI: 0.987–0.999) and acceptable performance in the validation set (AUC = 0.722, 95% CI: 0.525–0.919).

**Conclusion:**

Integration of baseline gut microbiota and lipidomic data revealed specific pre-disease microbial–lipid signatures, including positive *Blautia*–sphingomyelin and negative *Bacteroides*–triglyceride associations. A multi-omics model improved prediction of incident MetS over single-omics models, supporting the potential of microbiota–metabolite panels for early risk detection.

## Introduction

The global prevalence of metabolic syndrome (MetS) has increased rapidly, emerging as a major public health concern (Ma et al., 2024, Wang S. et al., 2021). According to World Health Organization (WHO) estimates, approximately 20%–30% of adults worldwide are affected by MetS (Raya-Cano et al., 2022). MetS is characterized by a cluster of interrelated metabolic abnormalities, including central (abdominal) obesity, hyperglycemia, hypertension, and dyslipidemia (Huang, 2009; Saklayen, 2018). Beyond substantially compromising quality of life, MetS increases the risk of several chronic diseases (Lee et al., 2023; Devesa et al., 2023). Epidemiological studies have demonstrated that individuals with MetS have a two- to four-fold higher risk of cardiovascular disease (CVD) compared with the general population, and their risk of developing type 2 diabetes mellitus (T2DM) is five- to ten-fold greater (Mir et al., 2022; Mazidi et al., 2016; O’neill and O’driscoll, 2014).

Moreover, MetS frequently co-occurs with conditions such as nonalcoholic fatty liver disease (NAFLD), sleep apnea–hypopnea syndrome (SAHS), and polycystic ovary syndrome (PCOS), further increasing disease burden and healthcare costs (Hall et al., 2024; Gao et al., 2024; Hartstra et al., 2015; Secchiero et al., 2024; Yu et al., 2023). Given these substantial risks, improved understanding of MetS pathogenesis is essential for effective prevention and management. The gut microbiota, a complex and dynamic microbial ecosystem, plays a central role in maintaining host metabolic homeostasis (Wang et al., 2020).

Accumulating evidence indicates that gut microbiota dysbiosis contributes to the onset and progression of MetS (Zeng et al., 2020; Sheng et al., 2022). This occurs largely through production of metabolites such as bile acids, short-chain fatty acids (SCFAs), and other bioactive molecules that influence host energy balance and lipid metabolism (Zhao et al., 2024). Alterations in microbial composition may therefore reshape metabolic activity, modify host metabolomic profiles, and promote MetS-related metabolic disturbances through multiple biological pathways (Canto-Osorio et al., 2020; Wang et al., 2018). Lipids are fundamental components of cell structure and function, serving critical roles in energy storage, membrane integrity, and signaling (Petrenko et al., 2023). Dysregulated lipid metabolism is a hallmark of MetS, and lipidomic profiling enables comprehensive examination of the lipid species, concentrations, and alterations underlying the disease process (Mocciaro et al., 2022).

Although growing evidence implicates gut microbiota in MetS pathophysiology, multi-omic interplay between gut microbiota and host lipid metabolism in the progression to MetS still warrants further elucidation. Most previous studies have examined gut microbial profiles or metabolic changes in isolation, rather than linking microbial composition, metabolic pathways, and lipid remodeling within a unified framework. Recent multi-omics studies have begun to address this gap, revealing coordinated changes between the microbiome and lipidomic profiles that contribute to metabolic dysfunction (Du et al., 2025, Zhang et al., 2025, Wang et al., 2023). These integrative approaches underscore the need to examine MetS from a systems-level perspective rather than as isolated biological domains.

Accordingly, this study aimed to investigate the interplay between gut microbiota and lipid metabolism in MetS by integrating microbial profiling with lipidomic analysis. By comparing individuals with MetS and healthy controls, we sought to identify key microbial taxa and lipid species associated with the condition and to examine potential microbial-metabolic pathways involved in lipid dysregulation and MetS development. We anticipate that this integrative approach will clarify microbiota-lipid relationships and identify potential targets for early intervention or therapy.

## Materials and methods

### Study population

This study was approved by the Ethics Committee of the First Affiliated Hospital of Zhengzhou University (Approval No. 2018-KY-90). All participants provided written informed consent after being informed of the study aims and procedures.

We conducted a nested case–control study within a prospective cohort of adults who underwent routine health examinations at the Health Management Center of the First Affiliated Hospital of Zhengzhou University between January 2018 and February 2019. All participants were free of metabolic syndrome (MetS) at baseline and were followed annually from March 2019 to December 2023. Individuals who developed MetS during follow-up were classified as incident cases; those who remained metabolically healthy served as controls.

Participants were screened according to predefined inclusion and exclusion criteria. Exclusion criteria were: (1) gastrointestinal disorders such as diarrhea, irritable bowel syndrome, or inflammatory bowel disease; (2) use of antibiotics, probiotics, gastric mucosal protectants, or other microbiota-modifying agents within the previous 3 months; (3) malignant tumors or severe diseases affecting major organs; (4) pregnancy or lactation; or (5) incomplete clinical or laboratory data.

To minimize potential confounding, cases and controls were matched 1:1 by age and sex. Key metabolic parameters related to MetS components-including systolic blood pressure (SBP), diastolic blood pressure (DBP), waist circumference (WC), fasting blood glucose (FBG), triglycerides (TG), and high-density lipoprotein cholesterol (HDL-C)-were also considered during matching to ensure baseline comparability between groups. Matching based on the abnormal rates of MetS components (defined as the proportion of participants with abnormal values for each component at baseline) was conducted to prevent baseline non-MetS participants from showing intergroup differences in MetS components.

### Diagnostic criteria for MetS

MetS was diagnosed according to the Guidelines for the Prevention and Treatment of Diabetes in China (2024), issued by the Chinese Diabetes Society (CDS) (中华医学会糖尿病学分会, 2025). Participants were classified as having MetS if they met at least three of the following five criteria:

1. Central obesity: WC ≥ 90 cm in men or ≥ 85 cm in women;
2. Hyperglycemia: FBG ≥ 6.1 mmol/L, or 2-h post-load glucose ≥ 7.8 mmol/L, and/or previously diagnosed type 2 diabetes mellitus under treatment;
3. Hypertension: SBP ≥ 130 mmHg or DBP ≥ 85 mmHg (1 mmHg = 0.133 kPa), or ongoing treatment for hypertension;
4. Hypertriglyceridemia: TG ≥ 1.70 mmol/L;
5. Low HDL-C: HDL-C < 1.04 mmol/L.

### Data collection and clinical measurements

Demographic and lifestyle information, medical history, and medication use were collected through structured questionnaires administered by trained nursing staff. Physical examinations were performed under standardized conditions. Fasting venous blood samples were collected in the morning after at least 8 hours of fasting. Hematological parameters, including hemoglobin (HGB) and white blood cell (WBC) count, were measured using a five-part differential hematology analyzer (BC-6800Plus, Mindray, China). Biochemical analyses-including glucose, lipid profiles, and liver and kidney function tests-were performed using an automated biochemical analyzer (Cobas 8000, Roche, Germany). Lipid parameters included TG, total cholesterol (TC), HDL-C, and low-density lipoprotein cholesterol (LDL-C). Liver function was assessed by measuring alanine aminotransferase (ALT), aspartate aminotransferase (AST), and *γ*-glutamyl transferase (GGT). Kidney function indices included serum creatinine (CRE), blood urea nitrogen (BUN), uric acid (SUA), and estimated glomerular filtration rate (eGFR). Glycated hemoglobin (HbA1c) was measured using an automated hemoglobin analyzer (HA-8180, Arkray, Japan).

### Metagenomic sequencing and functional annotation

At baseline, defined as the time of the initial health examination prior to the follow-up period for incident MetS, fresh fecal samples were collected immediately after defecation from the inner portion of the stool and stored at −80 °C until analysis. Microbial DNA was extracted using a commercial fecal DNA extraction kit (Magan Biotech, Guangzhou, China) according to the manufacturer’s instructions. We used shotgun metagenomic sequencing to construct the Whole Genome Sequencing library. Library preparation comprised DNA quality assessment, fragment selection (200–400 bp), end repair, adapter ligation, PCR amplification, and magnetic bead purification. Sequencing was performed on the MGI-SEQ-2000 platform (BGI, China). Raw reads were quality-filtered to remove adapters, low-quality bases (≥50% bases with Q ≤ 5), and sequences containing more than 10% ambiguous nucleotides.

Taxonomic profiling was performed using MetaPhlAn2 (Truong et al., 2015), which estimates relative abundance of microbial taxa across hierarchical levels based on clade-specific marker genes. Functional annotation was conducted using the HUMAnN2 pipeline in conjunction with the NCBI database and the Human Microbiome Project Unified Metabolic Analysis Network for nonredundant gene family annotation and pathway reconstruction (Fang et al., 2018; Li et al., 2021).

### Serum lipidomics analysis

Fasting serum samples were transported on dry ice to BGI Tech Solutions Co., Ltd. (Shenzhen, China) for lipidomic analysis. The process for extracting metabolites is as follows: Firstly, 100 μL of each sample was added to a 96-well plate. Secondly, Fetal bovine serum was added to the first well, followed by one well of fetal bovine serum for every 10 samples. Thirdly, 300 μL of pre-cooled isopropanol (containing an internal standard) was added, the plate was sealed, and shaken for 1 min, then placed in a −20 °C freezer for overnight sedimentation. Fourth, The samples were centrifuged at 4 °C for 30 min at a speed of 4,000 revolutions per minute. Finally, 10 μL of each sample was taken for mixing with QC (Quality Control), and then another 150 μL was added to a new 96-well plate.

Lipid extraction, separation, and detection were performed using an ultra-performance liquid chromatography (UPLC) system (Waters 2777C, USA) coupled to a Q Exactive HF high-resolution mass spectrometer (Thermo Fisher Scientific, USA). Chromatographic separation was performed on a Waters CSH C18 column (2.1 × 100 mm, 1.7 μm) at a constant temperature of 55 °C. Mass spectrometric detection was carried out using a Q Active HF mass spectrometer (Thermo Fisher Scientific), with the spray voltage set to 3.80 kV in positive-ion mode and 3.20 kV in negative-ion mode. Both MS1 and MS2 data were acquired for metabolite profiling.

The offline data generated by the mass spectrometer were imported into LipidSearch v.4.1 (Thermo Fisher Scientific, USA) software for metabolite signal extraction and identification. This software provides information including peak areas and identification results for each metabolite. Subsequently, metaX was applied to further process the extracted data to obtain compound information and quantitative values suitable for formal analysis. The identification results of these metabolites were annotated using the Human Metabolome Database (HMDB, http://www.hmdb.ca). Lipid species were annotated based on accurate mass, retention time, and MS/MS fragmentation patterns.

To eliminate systematic errors, the following procedures were performed: lipid molecules with missing values exceeding 50% in quality control (QC) samples and 80% in experimental samples were removed; missing values in the dataset were imputed using the K-Nearest Neighbor (KNN) algorithm, and data were normalized using the Probabilistic Quotient Normalization (PQN [8]) method; batch effects in the experimental data were corrected using QC-RLSC [9] (quality control-based robust LOESS signal correction with local polynomial regression fitting). Finally, lipid molecules with a coefficient of variation (CV) of relative peak areas exceeding 30% across all QC samples were excluded from further analysis.

### Statistical analysis

All statistical analyses were performed using R (version 4.2.2), with two-tailed *p* < 0.05 considered statistically significant. Categorical variables were expressed as counts and percentages and compared using the McNemar’s test. Continuous variables were expressed as mean ± standard deviation (SD) or median (interquartile range) according to distribution, which was assessed using the Shapiro–Wilk test. Between-group differences were evaluated using paired *t*-test or the Wilcoxon signed-rank test as appropriate. Microbial *α*-diversity and *β*-diversity were calculated to assess within-sample and between-sample diversity, respectively. Principal component analysis (PCA) and orthogonal partial least squares discriminant analysis (OPLS-DA) were applied for pattern recognition and group discrimination. Differential taxa were identified using STAMP software with Welch’s t-test. For lipidomic data, OPLS-DA was used to identify discriminative lipid species, with model robustness evaluated through permutation testing and cross-validation. Differential lipids were defined by variable importance in projection (VIP > 1.0) combined with univariate *p* < 0.05. Spearman’s rank correlation analysis was used to examine associations among differential microbes, lipid metabolites, and clinical variables.

### Predictive modeling and multi-omics integration

**Figure 1:**
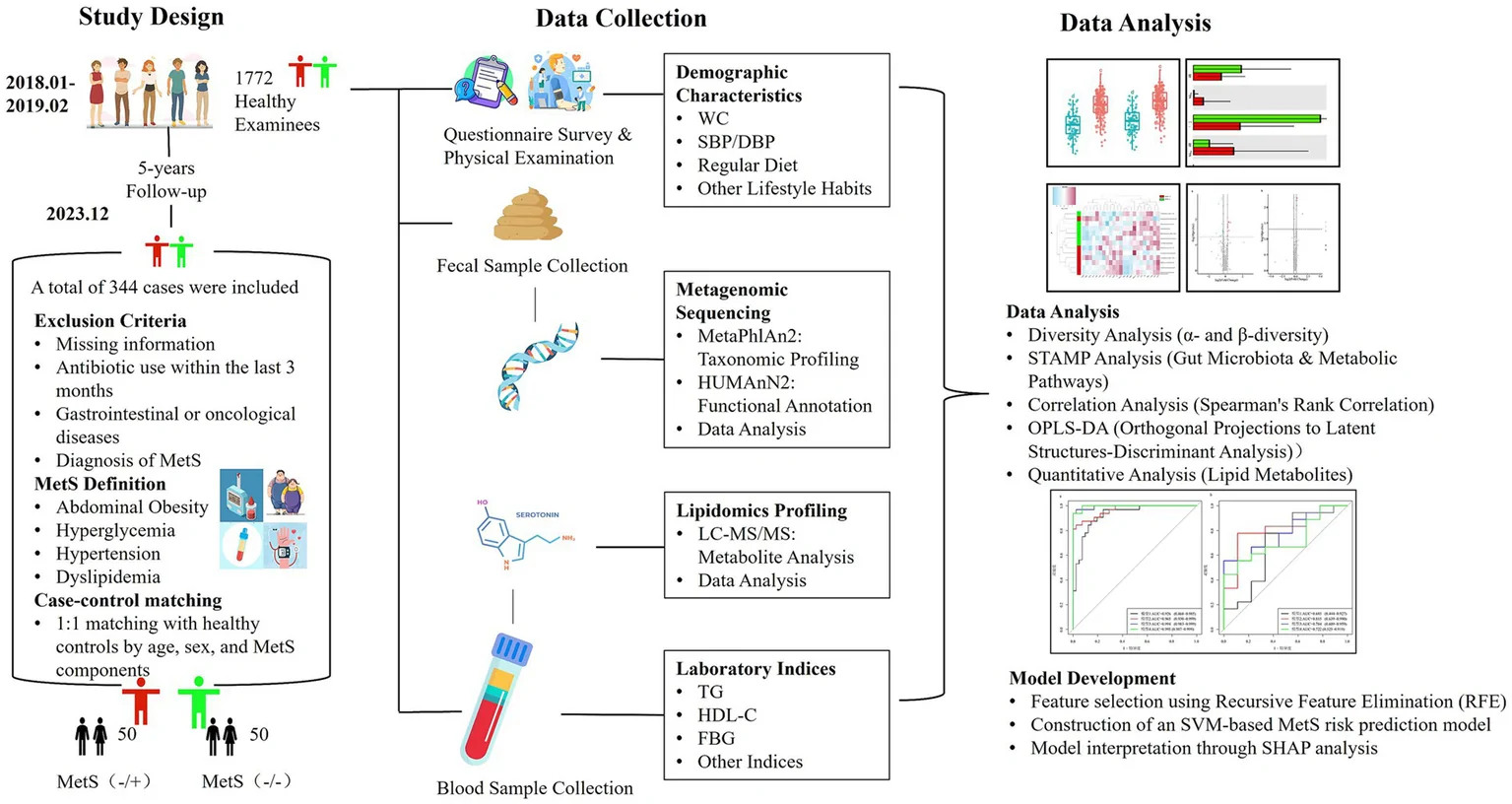
Research design flowchart. Participants were followed up from 2019 to 2023 to identify incident MetS cases.

To evaluate the predictive contribution of microbial and lipidomic features, recursive feature elimination (RFE) was applied to select the top 15 predictors. A support vector machine (SVM) classifier was constructed using the combined feature set. Data were randomly partitioned into training (70%) and testing (30%) sets. Model parameters were optimized using grid search with five-fold cross-validation to minimize overfitting. Model performance was assessed using the area under the receiver operating characteristic curve (AUC), accuracy, sensitivity, and specificity. The complete analytical workflow is presented in Figure 1. Previous studies have demonstrated that machine learning–based integration of multi-omics data can improve disease prediction (Wu et al., 2025). Futhermore, we made a two-dimensional projection of the samples based on the top discriminating features identified by the recursive feature elimination and SHAP analysis.

## Results

### Baseline characteristics of study participants

Of 1,772 individuals initially screened, 344 met the inclusion criteria. After 1:1 matching based on age, sex, and abnormal rates of MetS components, 100 participants (50 with MetS and 50 healthy controls) were included in the analysis. As shown in Table 1, demographic characteristics did not differ significantly between groups (*p* > 0.05). However, HDL-C levels were significantly lower in participants with MetS compared with controls (*p* < 0.05). The distribution of abnormal MetS components showed no significant between-group difference (Supplementary Table S1). These results confirm successful matching and baseline comparability between the MetS and control groups aside from metabolic abnormalities.

**Table 1:** Comparison of demographic baseline characteristics.

Characteristics	Control	Case	*p value*
Age	41.00 (37.25, 50.00)	44.00 (38.00,49.50)	0.160
Sex	Male: 44 (88.00%) Female: 6 (12.00%)	Male: 44 (88.00%) Female: 6 (12.00%)	0.999
WC (cm)	88.88 ± 7.09	90.13 ± 7.05	0.189
SBP (mmHg)	131.02 ± 12.67	131.72 ± 14.39	0.673
DBP (mmHg)	82.05 ± 10.57	82.34 ± 10.50	0.833
Height (cm)	171.43 ± 7.00	171.04 ± 7.73	0.710
Weight(kg)	75.27 ± 9.32	77.70 ± 9.97	0.097
BMI (kg/m^2^)	25.57 ± 2.50	26.53 ± 2.69	0.031
Regular diet	No: 7 (14.00%) Yes: 43 (86.00%)	No: 1 (2.00%) Yes: 49 (98.00%)	0.077
Dietary habit	Vegetarian: 6 (12.00%) Meat: 39 (78.00%) Mixed: 5 (10.00%)	Vegetarian: 2 (4.00%) Meat: 36 (72.00%) Mixed: 12 (24.00%)	0.068
Whole grain	No: 5 (10.00%) Yes: 45 (90.00%)	No: 6 (12.00%) Yes: 44 (88.00%)	0.999
Smoking	No: 36 (72.00%) Yes: 14 (28.00%)	No: 38 (76.00%) Yes: 12 (24.00%)	0.814
Drinking	No: 18 (36.00%) Yes: 32 (64.00%)	No: 24 (48.00%) Yes: 26 (52.00%)	0.307
Exercise	Never: 7 (14.00%) Occasionally: 23 (46.00%) Regularly: 20 (40.00%)	Never: 4 (8.00%) Occasionally: 31 (62.00%) Regularly: 15 (30.00%)	0.740
WBC (×10^9^/L)	6.55 ± 1.65	6.29 ± 1.59	0.336
RBC (×10^9^/L)	4.96 ± 0.34	5.01 ± 0.32	0.330
HGB (g/L)	153.52 ± 10.80	156.04 ± 13.00	0.191
PLT (×10^9^/L)	242.56 ± 57.23	239.98 ± 50.29	0.803
ALT (U/L)	28.08 ± 15.47	25.24 ± 12.18	0.299
AST (U/L)	22.54 ± 7.42	21.52 ± 7.35	0.441
GGT (U/L)	26.00 (18.25, 44.50)	29.00 (21.25, 44.00)	0.506
ALP (U/L)	72.86 ± 15.20	71.60 ± 15.86	0.667
TP (g/L)	75.90 (72.98, 78.70)	76.15 (72.48, 78.95)	0.843
ALB (g/L)	49.22 ± 2.75	49.28 ± 1.87	0.904
GLB (g/L)	26.42 ± 3.85	26.64 ± 3.83	0.798
TBIL (μmol/L)	9.71 (8.13, 11.80)	11.38 (9.69, 13.85)	0.051
DBIL (μmol/L)	4.42 (3.68, 5.35)	4.59 (3.89, 5.99)	0.183
IBIL (μmol/L)	5.35 (4.53, 6.75)	6.65 (5.48, 8.10)	0.031
BUN (mmol/L)	4.94 ± 1.19	4.72 ± 1.33	0.342
CRE (μmol/L)	76.76 ± 10.37	78.98 ± 12.68	0.245
SUA (μmol/L)	341.78 ± 77.06	353.72 ± 82.28	0.355
eGFR [ml/(min*1.73m^2^)]	101.95 ± 10.27	99.66 ± 12.36	0.307
TC (mmol/L)	4.79 (4.31, 5.35)	4.89 (4.23, 5.36)	0.602
TG (mmol/L)	1.39 (1.07, 1.93)	1.51 (1.21, 2.22)	0.077
HDL-C (mmol/L)	1.28 ± 0.26	1.40 ± 0.34	0.011
LDL-C (mmol/L)	3.02 (2.62, 3.36)	3.01 (2.57, 3.29)	0.602
FBG (mmol/L)	5.21 ± 0.50	5.12 ± 0.42	0.329

WC, waist circumference; SBP, systolic blood pressure; DBP, diastolic blood pressure; BMI, body mass index; WBC, white blood cell; RBC, red blood cell; HGB, hemoglobin; PLT, platelet count; ALT, glutamic pyruvic transaminase; AST, glutamic oxaloacetic transaminase; GGT, gamma-glutamyl transferase; ALP, alkaline phosphatase; TP, total protein; ALB, albumin; GLB, globulin; TBIL, total bilirubin; DBIL, direct bilirubin; IBIL, indirect bilirubin; BUN, blood urea nitrogen; CRE, creatinine; SUA, serum uric acid; eGFR, estimated glomerular filtration rate; TC, total cholesterol; TG, triglyceride; HDL-C, high-density lipoprotein cholesterol; LDL-C, low-density lipoprotein cholesterol; FBG, fasting blood glucose. Paired *t*-test, Wilcoxon signed-rank test and McNemar’s test were used to compare the differences between the control group (*n* = 50) and the MetS group (*n* = 50).

### Gut microbiota diversity

At the species level, *α*-diversity indices—including Shannon, observed species (Obs), Simpson, and Gini indices did not differ significantly between groups (*p* > 0.05; Supplementary Figure S1). Similarly, *β*-diversity assessed by principal coordinates analysis (PCoA) revealed no apparent clustering separation (Supplementary Figure S2), indicating comparable overall microbial community structures. Although no global differences were detected, subsequent analyses revealed subtle taxonomic and functional shifts between the MetS and control groups.

### Differential gut microbiota and metabolic pathways

**Figure 2:**
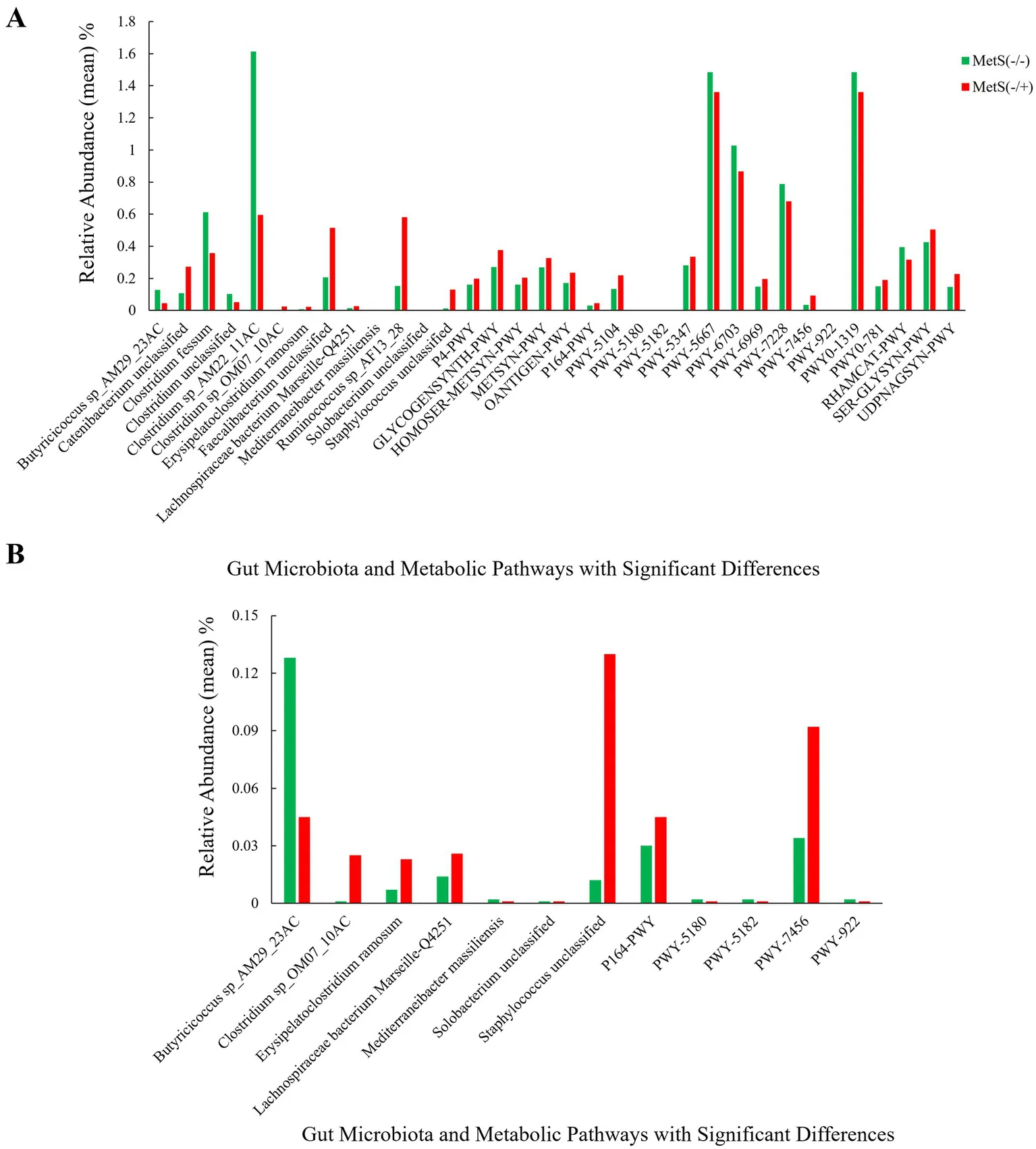
Differences in gut microbiota and metabolic pathways. MetS(−/−): The control group; MetS(−/+): The case group. **(a)** The relative abundances of the gut microbiota and metabolic pathways with significant differences. **(b)** The relative abundances of those entries that appear to be zero in both groups.

Using STAMP, we identified 13 microbial species and 13 metabolic pathways that differed significantly between groups (*p* < 0.05). Seven species and eight pathways were more abundant in participants with MetS, whereas six species were relatively depleted (Supplementary Figures S3, S4). Relative abundances of these taxa and their corresponding functional pathways are summarized in Figure 2 and Supplementary Table S3, with detailed annotations in Supplementary Tables S4, S5. Pathways enriched in the MetS group were primarily involved in carbohydrate and lipid metabolism, whereas several amino acid-related pathways were reduced. These observations suggest that gut microbial dysbiosis in MetS may involve functional reprogramming toward energy and lipid utilization, consistent with recent multi-omics findings (Du et al., 2025; Zhang et al., 2025; Wang et al., 2023).

### Lipidomic profiles in MetS

**Figure 3:**
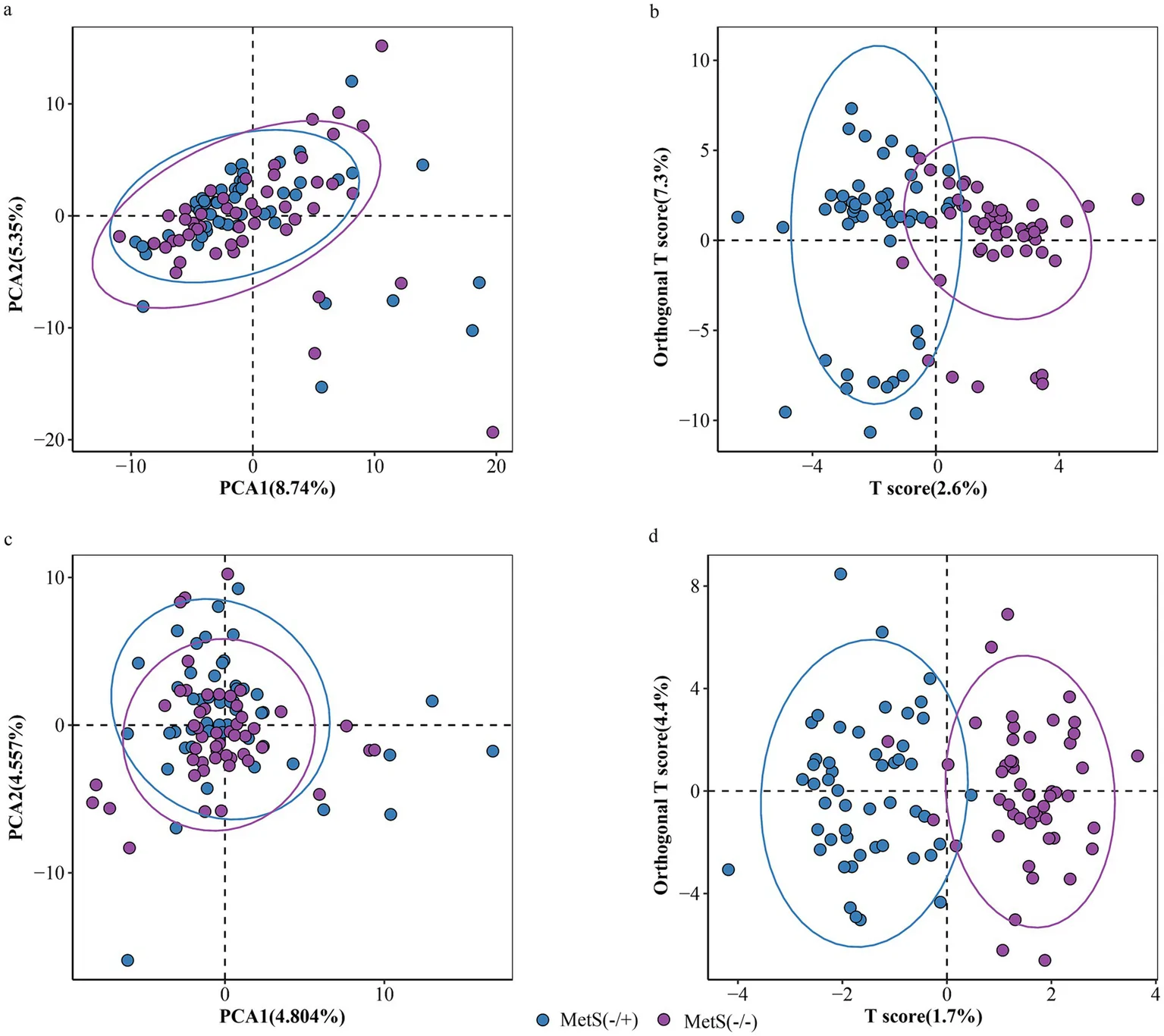
PCA and OPLS-DA score plots between MetS and control groups. **(a,b)** PCA and OPLS-DA score plots under the positive ion mode; **(c,d)** PCA and OPLS-DA score plots under the negative ion mode. Each point represents one sample, colored according to group, and ellipses indicate the 95% confidence interval (95% CI).

Principal component analysis (PCA) was applied in both positive and negative ion modes to assess overall lipidomic variation. As shown in Figures 3a,c, the two groups overlapped considerably, with only a few samples separating from the main cluster, indicating broadly similar lipid profiles. In contrast, orthogonal partial least squares discriminant analysis (OPLS-DA) revealed clearer, though partial, separation between groups in both modes (Figures 3b,d), suggesting subtle metabolic differences in individuals with MetS. Differential metabolites were identified based on tandem mass spectrometry data and database annotation using thresholds of VIP ≥ 1.0, fold change (FC) ≥ 1.2 or ≤ 0.83, and adjusted *p* < 0.05. Fifteen lipid species met these criteria (Table 2), with relative abundances shown in Supplementary Figure S5. In positive ion mode, 11 lipid species were elevated in MetS; in negative ion mode, four lipids showed higher relative levels (Supplementary Figure S6). Most alterations involved triglycerides (TG), phosphatidylcholines (PC), and ceramides (CerG1), which accounted for the majority of lipid changes associated with MetS. This lipidomic pattern characterized by TG and ceramide elevation alongside PC reduction is consistent with metabolic lipid remodeling reported in previous MetS cohorts (Du et al., 2025; Zhang et al., 2025; Wang et al., 2023).

**Table 2:** Names and classification of significantly differential metabolites.

Ion modes	Metabolite ID	Name	*VIP*	State	Classification
Positive	10.79_888.802956357143	TG(17:0/18:1(9Z)/18:2(9Z,12Z))	1.353	Down	GL
Positive	10.91_876.803572371429	TG(16:0/18:1(9Z)/18:1(9Z))	2.675	Down	GL
Positive	11.29_984.895596304762	TG(18:1(11Z)/18:2(9Z,12Z)/24:1(15Z))	2.206	Down	GL
Positive	7.78_608.525011812785	DG(16:1/18:2)	3.219	Up	GL
Positive	7.88_634.541168196347	DG(18:2/18:2)	2.492	Up	GL
Positive	8.11_660.556387	DG(20:3(5Z,8Z,11Z)/18:2(9Z,12Z)/0:0)	2.447	Up	GL
Positive	8.58_810.683108152381	CerG1(d18:1/24:1)	1.089	Down	SP
Positive	0.92_468.308888844749	LysoPC(14:0/0:0)	2.962	Up	GP
Positive	10.54_664.603782528571	ChE(18:3)	1.324	Down	ST
Positive	11.31_888.837983438095	TG(18:0e/18:1/18:2)	1.091	Up	GL
Positive	5.83_904.590701289474	PI(18:0/20:4(5Z,8Z,11Z,14Z))	2.289	Down	GP
Positive	8.35_624.556163027397	DG(17:0/18:2(9Z,12Z)/0:0)	2.688	Up	GL
Positive	5.24_740.558934027397	PC(34:3p)	1.820	Down	GP
Positive	6.5_716.522624931507	PC(31:2)	1.145	Up	GP
Positive	6.52_748.527596486239	PC(35:6p)	2.255	Up	GP
Positive	6.65_844.621494242009	PC(42:7p)	1.401	Up	GP
Positive	7.34_726.543282082192	PE(36:4e)	1.139	Down	GP
Positive	7.88_776.558890730594	PC(37:6p)	3.116	Down	GP
Positive	8_826.632043766234	PC(39:3)	2.108	Down	GP
Positive	8.04_759.638173607306	SM(d38:1)	1.597	Down	SP
Positive	8.09_833.65135139726	SM(d44:6)	2.406	Down	SP
Positive	8.65_801.686207762557	SM(d41:1)	1.214	Down	SP
Positive	8.76_834.634736385714	PE(42:2p)	2.032	Up	GP
Positive	9.04_804.684156152381	PC(38:0e)	1.856	Down	GP
Negative	0.8_619.288891931818	LPI(20:4)	2.701	Up	GP
Negative	6.54_838.560369880952	PS(18:0/22:4(7Z,10Z,13Z,16Z))	2.751	Up	GP
Negative	6.75_774.544811957143	PE(18:0p/22:6)	2.400	Down	GP
Negative	7.96_752.560399157407	PE(18:0e/20:4)	1.580	Down	GP
Negative	4.78_801.543792509259	PA(26:5/18:2)	1.903	Up	GP
Negative	6.52_808.513224319048	PS(18:1(9Z)/20:4(5Z,8Z,11Z,14Z))	2.891	Up	GP
Negative	7.43_830.591725462963	PS(15:0/24:1(15Z))	1.801	Up	GP

Metabolite ID: A unique metabolite index composed of retention time and neutral mass, used to designate a specific metabolite. State: Representing the upregulated or downregulated expression levels in the context of MetS. GP, Glycerophospholipids; GL, Glycerolipids; SP, Sphingolipids; ST, Sterol Lipids.

### Correlations between gut microbiota and clinical indicators

**Figure 4:**
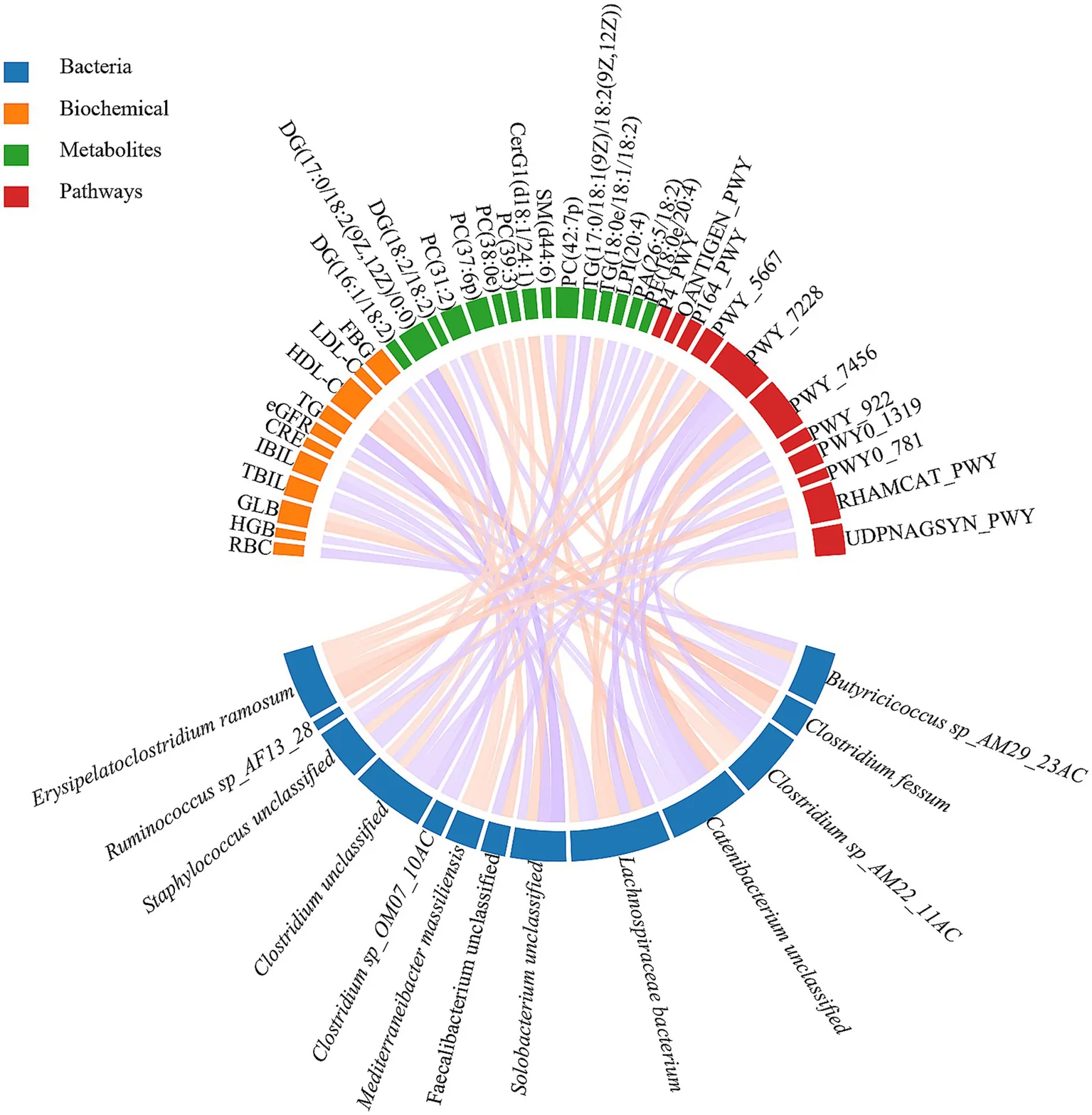
Correlation analysis between differential gut microbiota and other characteristics. Only variables showing significant correlations (*p* < 0.05) between differential bacteria, biochemical indicators, metabolites, and metabolic pathways are displayed. Connecting lines indicate correlations, with orange representing positive correlations and purple representing negative correlations; color intensity reflects correlation strength. Outer colored blocks represent different entities, including bacteria, biochemical indicators, metabolites, and metabolic pathways.

Spearman’s rank correlation analysis revealed significant associations between differential microbial taxa and clinical indicators (Figure 4). Clostridium sp._AM22_11AC correlated positively with serum creatinine (CRE) and globulin (GLB) but negatively with estimated glomerular filtration rate (eGFR). Clostridium fessum and Faecalibacterium unclassified were positively associated with HDL-C, whereas Mediterraneibacter massiliensis was associated with higher fasting blood glucose (FBG) and LDL-C. In contrast, Solobacterium unclassified was inversely associated with triglycerides (TG), and Butyricicoccus sp._AM29_23AC showed negative associations with hemoglobin (HGB) and red blood cell count (RBC). These findings indicate that specific gut microbial alterations are associated with distinct metabolic and hematologic characteristics in MetS.

### Associations between microbial taxa and metabolic pathways

Examination of microbial taxa in relation to functional metabolic pathways revealed that Erysipelatoclostridium ramosum showed positive associations with six pathways, whereas Catenibacterium unclassified exhibited both positive and negative correlations across several pathways (Figure 4). These observations suggest that individual gut microbes may influence host metabolism through specific functional routes rather than broad community level shifts.

### Microbiota–lipid metabolite interactions

Correlation analysis between differential microbial taxa and lipid metabolites revealed several notable associations (Figure 4). Lachnospiraceae bacterium Marseille-Q4251 showed positive correlations with CerG1 (d18:1/24:1) and PC (31:2), whereas Clostridium sp._AM22_11AC was positively associated with TG (18:0e/18:1/18:2) but negatively associated with PC (31:2). Collectively, these results suggest potential interplay between gut microbial composition and host lipid metabolism in MetS, consistent with recent reports of cross-talk between the gut microbiome and lipidomic networks (Du et al., 2025; Zhang et al., 2025; Wang et al., 2023). All correlations were calculated using Spearman’s method (*p* < 0.05); detailed coefficients are presented in Supplementary Table S6.

### Prediction model

#### Feature selection and model construction

To evaluate the contribution of microbial and lipidomic alterations to MetS incidence risk prediction, we applied recursive feature elimination (RFE) to select the top 15 predictive variables. These features were used to construct a support vector machine (SVM) model. For comparison, simpler models were constructed using only demographic information or standard laboratory indicators (Supplementary Figure S10).

#### Model performance

**Figure 5:**
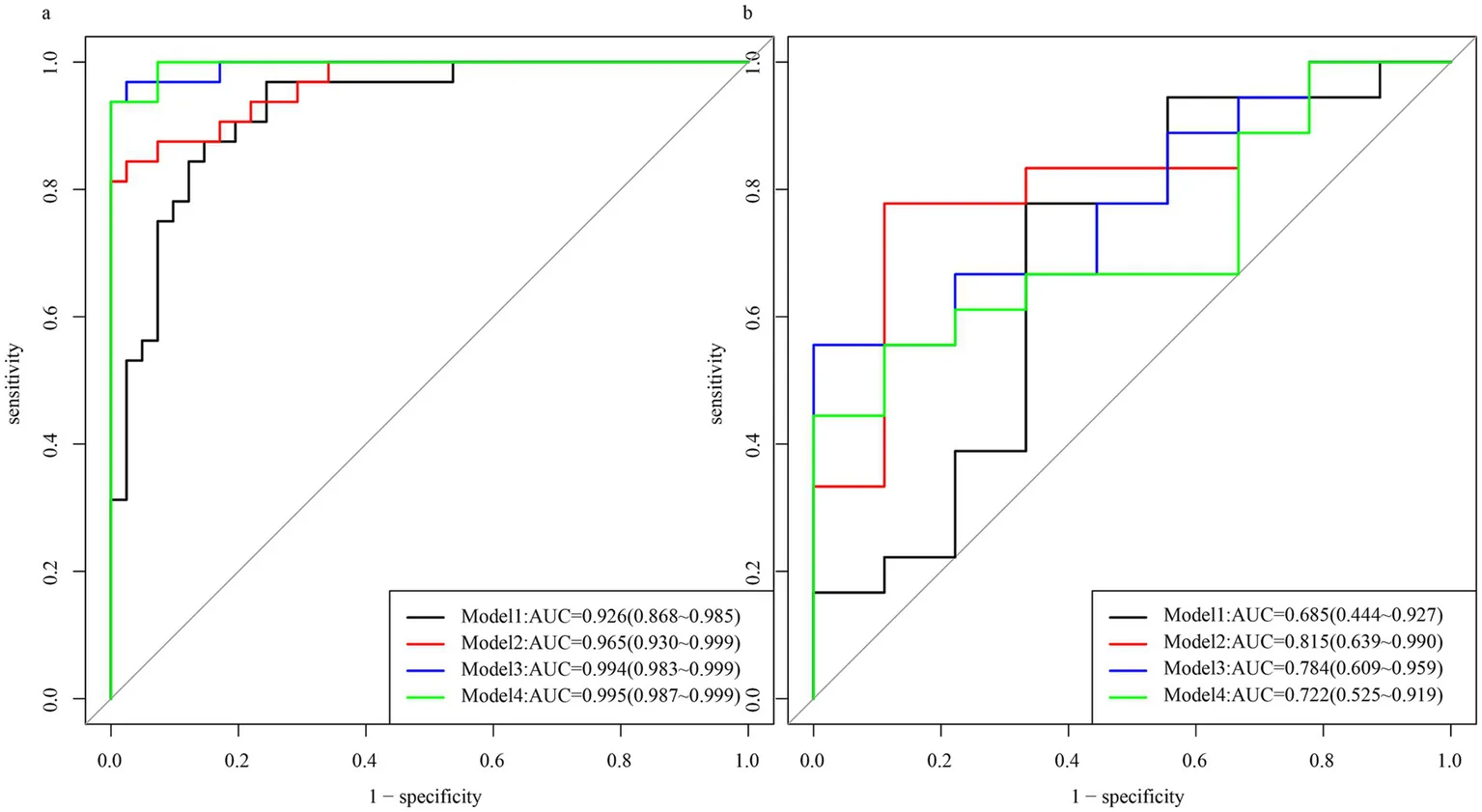
ROC curves of MetS risk prediction models. Model 1 was constructed using laboratory indicators and general demographic information. Model 2 extended Model 1 by incorporating differential gut microbiota. Model 3 extended Model 1 by incorporating differential metabolites. Model 4 incorporated both differential gut microbiota and differential metabolites based on Model 1. **(a)** Training set; **(b)** Test set.

Model performance was evaluated using receiver operating characteristic (ROC) curves (Figure 5) and classification metrics including accuracy, precision, recall, F1 score, and the Youden index (Supplementary Table S7). Among four tested models, the integrated model combining microbial and lipidomic features (Model 4) achieved the highest discriminative ability, with an AUC of 0.995 (95% CI: 0.987–0.999) in the training set and 0.722 (95% CI: 0.525–0.919) in the validation set. Models based solely on demographic or laboratory variables showed lower performance. These findings suggest that integrating gut microbiota and lipidomic profiles can improve predictive accuracy for identifying individuals at the risk of MetS (Wu et al., 2025).

### Feature interpretation via SHAP analysis

To understand how individual features contributed to model predictions, we applied SHAP (Shapley Additive Explanations) analysis. Lower abundances of Erysipelatoclostridium ramosum and Staphylococcus unclassified were associated with higher predicted MetS risk, whereas reduced Butyricicoccus sp._AM29_23AC abundance was associated with lower predicted risk (Supplementary Figure S11). These findings suggest that certain gut taxa play distinct roles within the predictive model and may be biologically relevant to MetS development.

## Discussion

Metabolic syndrome (MetS) is a multifactorial disorder involving several interlinked mechanisms, including insulin resistance (IR), chronic inflammation, and oxidative stress. Our nested case–control design enabled temporal assessment of incident MetS cases within a well-characterized prospective cohort. In IR, adipocytes, muscle cells, and hepatocytes exhibit diminished insulin sensitivity, resulting in impaired glucose regulation and hyperglycemia (Huang, 2009; Brown and Walker, 2016). Chronic inflammation further disrupts insulin signaling, promotes adipocyte differentiation and proliferation, and contributes to obesity while damaging vascular endothelial cells and accelerating atherosclerosis (Du et al., 2024; Lent-Schochet et al., 2019; Wan et al., 2024). Oxidative stress aggravates these disturbances by impairing cellular function, thereby amplifying IR, inflammation, and vascular injury (Lemos et al., 2023). The gut microbiota has emerged as a key regulator of host metabolism, with mounting evidence linking gut microbiota dysbiosis to MetS development through effects on energy metabolism, inflammatory responses, and insulin sensitivity (Stoeva et al., 2021; Sasidharan Pillai et al., 2024; D’Aversa et al., 2013). Alterations in microbial composition can modulate host metabolic pathways via microbial metabolites, thereby reshaping systemic metabolism. In the present study, we identified significant correlations between several microbial taxa and distinct metabolic pathways (Qin et al., 2021; Ussar et al., 2015), supporting the concept of a complex, integrated network connecting gut microbiota, metabolic pathways, and host metabolites in MetS (Bai et al., 2023; Chen and Devaraj, 2018).

Several microbial taxa identified in this study may contribute to metabolic disturbances characteristic of MetS. Erysipelatoclostridium ramosum was enriched in the MetS group; this bacterium has previously been detected at elevated levels in individuals with inflammatory bowel disease and is positively associated with body mass index (BMI), suggesting a potential role in promoting intestinal inflammation or enhancing nutrient absorption (Ning et al., 2023; Yang et al., 2024).

Similarly, Staphylococcus unclassified (phylum Firmicutes) may influence host immunity and insulin signaling. Previous studies have reported that *S. aureus* interferes with insulin receptor pathways and contributes to IR (Liu et al., 2018; Su et al., 2015). Although the specific Staphylococcus species in our cohort remains unidentified, its negative association with MetS risk is consistent with these earlier findings. In contrast, Clostridium unclassified taxa were enriched in the control group. Members of this genus have been associated with regulation of lipid absorption and body weight changes, suggesting that reduced abundance may contribute to lipid metabolic abnormalities and MetS development (Petersen et al., 2019). Beyond compositional changes, microbial metabolites such as lipopolysaccharide (LPS) can influence metabolic health by altering intestinal barrier integrity and eliciting subclinical inflammation (Bishehsari et al., 2020; Croci et al., 2021; Wang Y. et al., 2021).

Lipid metabolites identified in this study—including phosphatidylethanolamine (PE), triglycerides (TG), diacylglycerol (DG), and phosphatidylcholine (PC)-are closely associated with dyslipidemia and IR, both hallmark features of MetS (Denisenko et al., 2020; Šilhavý et al., 2023). Functional pathway enrichment indicated increased microbial carbohydrate, lipid, and biosynthetic activity in the MetS group. Such metabolic shifts may enhance DG utilization and impair insulin sensitivity (Semnani-Azad et al., 2024), providing mechanistic support for the observed associations between gut microbiota and host lipid metabolism. The combined microbial and metabolomic patterns are consistent with recent reports indicating that microbial dysbiosis contributes to altered lipid flux, increased oxidative stress, and proinflammatory signaling in MetS (Du et al., 2025; Zhang et al., 2025; Wang et al., 2023). The interplay between microbial activity and host metabolism may therefore represent a central mechanism underlying metabolic dysfunction.

Correlation analyses from this study allow several potential mechanisms to be postulated. First, overabundance of *E. ramosum* may promote LPS biosynthesis via the OANTIGEN-PWY pathway and, in the context of impaired intestinal permeability, elevate systemic inflammation, ultimately contributing to MetS. Second, elevated Staphylococcus unclassified may enhance carbohydrate degradation pathways such as mannan metabolism (PWY-7456), increasing energy absorption and influencing lipid metabolites such as PC(42:7p) and DG(18:2/18:2), potentially exacerbating dyslipidemia. Third, reduced Clostridium unclassified abundance may diminish protective metabolic functions, thereby affecting TG(17:0/18:1/18:2) and PE(18:0e/20:4) levels and promoting IR. These interactions illustrate how microbial metabolic activity can modulate systemic lipid balance and insulin sensitivity, providing plausible biological links between gut dysbiosis and MetS pathogenesis.

Integration of multi-dimensional data using support vector machine (SVM) and recursive feature elimination (RFE) improved predictive accuracy for MetS. Incorporating both differential microbial taxa and lipid metabolites enhanced classification performance compared with demographic or biochemical models alone, underscoring the predictive value of microbiota–lipid interactions. In the test set, the integrative Model 4 shows a numerically lower AUC than Models 2 and 3, likely due to overfitting on the training data given the higher dimensionality of combined features. When the dimensionality of variables increases without a proportional increase in sample size (e.g., when the number of variables exceeds one-tenth of the sample size), the data become sparse in the high-dimensional space, making it difficult for the model to capture the true underlying patterns. These findings suggest that integrative frameworks may serve as valuable tools for precision nutrition and personalized risk assessment in metabolic diseases. Such machine learning approaches could eventually support targeted therapeutic interventions by identifying high-risk individuals based on their gut–metabolic signatures.

Several limitations should be acknowledged. First, the relatively limited sample size may have constrained statistical power to detect subtle microbiota–metabolite associations. Second, although the nested case–control design provides temporal context within a prospective cohort, it limits causal inference regarding relationships among gut microbiota, lipid metabolites, and MetS. Third, despite strong internal performance, external validation of the machine learning model using an independent cohort was not performed. Finally, mechanistic validation experiments—such as targeted metabolite assays or microbial functional studies—were not conducted and warrant future investigation. Despite these limitations, our integrative multi-omics analysis provides valuable insights into the microbial–metabolic interplay underlying MetS.

## Conclusion

we applied a multi-omics framework combined with machine learning analysis to characterize differences in gut microbiota, metabolic pathways, and lipid metabolites between individuals with and without MetS. Our findings indicate that alterations in gut microbial composition and lipid metabolism are closely associated, forming an interconnected network that may contribute to MetS development. In particular, the positive correlation between *Blautia* abundance and sphingomyelin species, together with the negative correlation between *Bacteroides* and triglycerides, suggests a microbial–lipid interplay that could influence lipid homeostasis before MetS onset. Furthermore, enrichment of *Erysipelatoclostridium ramosum* and an unclassified *Staphylococcus* species, potentially linked to O-antigen biosynthesis (OANTIGEN-PWY) and PWY-7456 pathways, respectively, points toward microbial-driven inflammatory and metabolic disturbances. Conversely, the depletion of an unclassified *Clostridium* species may reflect diminished protective lipid-regulatory functions. A multi-omics machine learning model, incorporating selected microbiota and lipidomic features, achieved high discriminative performance for predicting incident MetS (training AUC = 0.995; validation AUC = 0.722), outperforming single-omics models. These findings identify specific microbial and lipid signatures that precede clinical MetS and may serve as candidate biomarkers for early risk stratification. Further mechanistic studies are warranted to validate the functional roles of the highlighted taxa and pathways in MetS development.

## Data Availability

The raw data supporting the conclusions of this article will be made available by the authors, without undue reservation. Our data has been deposited in Zenodo and is now publicly accessible. The DOI is: [10.5281/zenodo.21631068].
