# Supplementary material for: Integrated gut microbiome and serum lipidomics reveals microbial–lipid interactions for predicting incident metabolic syndrome: a nested case–control study
Source: Front Microbiol. 2026 Aug 6;17:1862738. doi: 10.3389/fmicb.2026.1862738 (PMC13490899; doi:10.3389/fmicb.2026.1862738)
Supplement: Supplementary file 1 [file Supplementary_file_1.docx]

Supplementary Material

1. Table S1. Comparison of MetS Components - *Page 2*.

2. Table S2. Comparison of Demographic Following Characteristics - *Pages 3*.

3. Table S3. The relative abundance of gut microbiota and with significant differences - *Pages 4-5*.

4. Table S4. Functional Classification of Metabolic Pathways - *Pages 6-8*.

5. Table S5. Full Names Corresponding to Pathway Codes - *Pages 9*.

6. Table S6. Correlation Coefficients between Gut Microbiota and Clinical Indicators, Metabolites and Pathways - *Pages 10-12*.

7. Table S7. Evaluation Indicators for the Test Set of the Prediction Model - *Pages 13*.

8. Figure S1. Comparison of α diversity - *Pages 14*.

9. Figure S2. Comparison of β diversity - *Pages 15*.

10. Figure S3. Differences in Gut Microbiota Abundance - *Pages 16*.

11. Figure S4. Differences in Gut Microbiota Metabolic Pathways - *Pages 17*.

12. Figure S5. Volcano Plots for Differential Metabolite Analysis - *Pages 18*.

13. Figure S6. Z-score Analysis of Differential Metabolite Content - *Pages 19*.

14. Figure S7. Correlation Heatmap of Gut Microbiota and Clinical Indicators - *Pages 20*.

15. Figure S8. Correlation Heatmap of Gut Microbiota and Metabolic Pathways - *Pages 21*.

16. Figure S9. Correlation Heatmap of Gut Microbiota and Metabolite - *Pages 22*.

17. Figure S10. Ranking of Feature Importance - *Pages 23*.

18. Figure S11. Feature Interpretation of the Prediction Model - *Pages 24*.

19. Figure S12. PCA projection showing group separation based on selected multi-omics features - *Pages 25*.

Table S1 Comparison of MetS Components

| Characteristics | Control | Case | *P* value |
| --- | --- | --- | --- |
| WC |  |  | 0.999 |
| No | 25 (50.00%) | 24 (48.00%) |  |
| Yes | 25 (50.00%) | 26 (52.00%) |  |
| SBP |  |  | 0.999 |
| No | 22 (44.00%) | 21 (42.00%) |  |
| Yes | 28 (56.00%) | 29 (58.00%) |  |
| DBP |  |  | 0.999 |
| No | 33 (66.00%) | 33 (66.00%) |  |
| Yes | 17 (34.00%) | 17 (34.00%) |  |
| TG |  |  | 0.999 |
| No | 31 (62.00%) | 32 (64.00%) |  |
| Yes | 19 (38.00%) | 18 (36.00%) |  |
| HDL-C |  |  | 0.480 |
| No | 44 (88.00%) | 46 (92.00%) |  |
| Yes | 6 (12.00%) | 4 (8.00%) |  |
| FBG |  |  | 0.999 |
| No | 50 (100.00%) | 49 (98.00%) |  |
| Yes | 0 (0.00%) | 1 (2.00%) |  |

WC: waist circumference; SBP: systolic blood pressure; DBP: diastolic blood pressure; TG: triglyceride; HDL-C: high-density lipoprotein-cholesterol; FBG: fasting blood glucose. McNemar's test were used to compare the differences between the control group (n=50) and the MetS group (n=50).

Table S2 Comparison of Demographic Following Characteristics

| Characteristics | Control | case | *P value* |
| --- | --- | --- | --- |
| Age | 46.00 (41.00, 51.75) | 45.50 (41.00, 53.75) | 0.042 |
| Sex | Male: 44 (88.00%) Female: 6 (12.00%) | Male: 44 (88.00%) Female: 6 (12.00%) | 0.999 |
| WC(cm) | 92.83 ± 6.45 | 90.02 ± 7.19 | 0.028 |
| SBP(mmHg) | 138.77 ± 14.76 | 128.64 ± 15.95 | <0.001 |
| DBP(mmHg) | 88.01 ± 9.91 | 79.16 ± 9.73 | <0.001 |
| Height(cm) | 169.97 ± 7.99 | 169.79 ± 7.14 | 0.877 |
| Weight(kg) | 79.27 ± 10.38 | 75.76 ± 9.04 | 0.032 |
| BMI(kg/m2) | 27.40 ± 2.84 | 26.27 ± 2.52 | 0.024 |
| WBC(×109/L) | 6.46 ± 1.53 | 6.27 ± 1.47 | 0.518 |
| RBC(×109/L) | 5.03 (4.88, 5.16) | 5.02 (4.73, 5.16) | 0.246 |
| HGB(g/L) | 156.14 ± 10.07 | 152.07 ± 10.19 | 0.025 |
| PLT(×109/L) | 245.32 ± 45.16 | 240.28 ± 55.20 | 0.618 |
| ALT(U/L) | 25.00 (18.25, 30.75) | 19.98 (16, 27.75.00) | 0.138 |
| AST(U/L) | 21.00 (19.00, 25.75) | 21.00 (17.22, 24.00) | 0.755 |
| GGT(U/L) | 31.00 (22.75, 40.00) | 27.20 (18.90, 39.50) | 0.351 |
| ALP(U/L) | 77.76 ± 19.63 | 74.95 ± 18.17 | 0.438 |
| TP(g/L) | 74.30 ± 3.68 | 73.73 ± 3.34 | 0.453 |
| ALB(g/L) | 48.03 ± 2.78 | 47.69 ± 2.55 | 0.525 |
| GLB(g/L) | 26.27 ± 3.52 | 26.02 ± 3.55 | 0.744 |
| TBIL(μmol/L) | 11.52 (8.78, 14.15) | 11.07 (8.82, 14.37) | 0.977 |
| DBIL(μmol/L) | 4.79 ± 1.65 | 4.78 ± 1.70 | 0.994 |
| IBIL(μmol/L) | 6.30 (4.93, 8.98) | 6.14 (4.95, 8.48) | 0.938 |
| BUN(mmol/L) | 4.82 ± 1.10 | 5.27 ± 1.20 | 0.068 |
| CRE(μmol/L) | 79.30 ± 10.50 | 76.31 ± 10.72 | 0.119 |
| SUA(μmol/L) | 359.52 ± 78.32 | 348.56 ± 78.10 | 0.468 |
| eGFR(ml/(min*1.73m²) | 97.87 ± 11.93 | 100.21 ± 11.34 | 0.3 |
| TC(mmol/L) | 5.02 ± 0.86 | 4.72 ± 0.93 | 0.093 |
| TG(mmol/L) | 2.10 (1.82, 2.57) | 1.32 (1.00, 1.95) | <0.001 |
| HDL-C(mmol/L) | 1.13 ± 0.25 | 1.29 ± 0.25 | <0.001 |
| LDL-C(mmol/L) | 3.11 ± 0.75 | 2.95 ± 0.83 | 0.3 |
| FBG(mmol/L) | 5.19 (4.89, 5.69) | 5.22 (4.98, 5.51) | 0.832 |

WC, waist circumference; SBP, systolic blood pressure; DBP, diastolic blood pressure; BMI, body mass index; WBC, white blood cell; RBC, red blood cell; HGB, hemoglobin; PLT, platelet count; ALT, glutamic pyruvic transaminase; AST, glutamic oxaloacetic transaminase; GGT, gamma-glutamyl transferase; ALP, alkaline phosphatase; TP, total protein; ALB, albumin; GLB, globulin; TBIL, total bilirubin; DBIL, direct bilirubin; IBIL, indirect bilirubin; BUN, blood urea nitrogen; CRE, creatinine; SUA, serum uric acid; eGFR, estimated glomerular filtration rate; TC, total cholesterol; TG, triglyceride; HDL-C, high-density lipoprotein cholesterol; LDL-C, low-density lipoprotein cholesterol; FBG, fasting blood glucose. paired t‑test, Wilcoxon signed‑rank test and McNemar's test were used to compare the differences between the control group (n=50) and the MetS group (n=50).

Table S3 The relative abundance of gut microbiota and with significant differences

| Taxonomic features | control | case | *P* value |
| --- | --- | --- | --- |
| *Butyricicoccus sp_AM29_23AC* | 0.128±0.268 | 0.045±0.076 | 0.042 |
| *Catenibacterium unclassified* | 0.107±0.289 | 0.272±0.486 | 0.044 |
| *Clostridium fessum* | 0.612±0.630 | 0.357±0.304 | 0.013 |
| *Clostridium unclassified* | 0.102±0.158 | 0.051±0.061 | 0.038 |
| *Clostridium sp_AM22_11AC* | 1.612±3.093 | 0.595±0.685 | 0.029 |
| *Clostridium sp_OM07_10AC* | 0.001±0.004 | 0.025±0.080 | 0.035 |
| *Erysipelatoclostridium ramosum* | 0.007±0.020 | 0.023±0.051 | 0.046 |
| *Faecalibacterium unclassified* | 0.206±0.305 | 0.514±0.949 | 0.035 |
| *Lachnospiraceae bacterium Marseille-Q4251* | 0.014±0.026 | 0.026±0.029 | 0.043 |
| *Mediterraneibacter massiliensis* | 0.002±0.006 | 0.001±0.001 | 0.041 |
| *Ruminococcus sp_AF13_28* | 0.153±0.225 | 0.580±1.375 | 0.037 |
| *Solobacterium unclassified* | 0.001±0.001 | 0.001±0.001 | 0.044 |
| *Staphylococcus unclassified* | 0.012±0.057 | 0.130±0.344 | 0.021 |
| P4-PWY | 0.160±0.097 | 0.199±0.098 | 0.047 |
| GLYCOGENSYNTH-PWY | 0.271±0.159 | 0.376±0.181 | 0.003 |
| HOMOSER-METSYN-PWY | 0.161±0.086 | 0.205±0.124 | 0.043 |
| METSYN-PWY | 0.269±0.119 | 0.327±0.151 | 0.039 |
| OANTIGEN-PWY | 0.172±0.117 | 0.235±0.150 | 0.023 |
| P164-PWY | 0.030±0.026 | 0.045±0.036 | 0.025 |
| PWY-5104 | 0.135±0.121 | 0.219±0.196 | 0.012 |
| PWY-5180 | 0.002±0.005 | 0.001±0.001 | 0.044 |
| PWY-5182 | 0.002±0.005 | 0.001±0.001 | 0.044 |
| PWY-5347 | 0.281±0.119 | 0.335±0.137 | 0.042 |
| PWY-5667 | 1.485±0.311 | 1.361±0.305 | 0.049 |
| PWY-6703 | 1.027±0.332 | 0.867±0.334 | 0.020 |
| PWY-6969 | 0.148±0.108 | 0.197±0.122 | 0.041 |
| PWY-7228 | 0.787±0.305 | 0.681±0.210 | 0.047 |
| PWY-7456 | 0.034±0.087 | 0.092±0.118 | 0.007 |
| PWY-922 | 0.002±0.002 | 0.001±0.001 | 0.029 |
| PWY0-1319 | 1.485±0.311 | 1.361±0.305 | 0.049 |
| PWY0-781 | 0.150±0.091 | 0.189±0.090 | 0.040 |
| RHAMCAT-PWY | 0.395±0.208 | 0.315±0.144 | 0.030 |
| SER-GLYSYN-PWY | 0.425±0.186 | 0.504±0.211 | 0.050 |
| UDPNAGSYN-PWY | 0.147±0.121 | 0.227±0.173 | 0.009 |

Table S4 Functional Classification of Metabolic Pathways

| Functional Classification | Pathway Code | group | Specific Function |
| --- | --- | --- | --- |
| Amino Acid Metabolism / Biosynthesis | P4-PWY | Case | It provides three amino acids (L-lysine, L-threonine, and L-methionine) for cells, which are used in various physiological processes such as protein synthesis and metabolic regulation. |
|  | HOMOSER-METSYN-PWY | Case | Some bacteria utilize organic sulfur through this pathway: they first catalyze the transsulfuration of O-succinyl-L-homoserine and L-cysteine to form L-cystathionine via a series of reactions, then convert it to L-homocysteine, and finally generate L-methionine through methylation. |
|  | METSYN-PWY | Case | It is involved in the synthesis process from L-homoserine to L-methionine, providing a basis for the intracellular supply of methionine and other metabolic reactions that use methionine as a raw material. |
|  | PWY-5104 | Case | It catalyzes the relevant reactions to produce L-isoleucine, which is used for protein synthesis and serves as an intermediate in certain metabolic pathways. |
|  | PWY-5347 | Case | It provides L-methionine for cells, which is used in protein synthesis to support cell growth, repair, and the normal operation of various physiological functions. |
|  | PWY0-781 | Case | Aspartate is a precursor for the synthesis of multiple amino acids, and this pathway involves processes such as the synthesis of aspartate and various related amino acids downstream of it. |
|  | SER-GLYSYN-PWY | Case | It provides cells with L-serine and glycine, two amino acids that play important roles in processes such as one-carbon unit metabolism and nucleotide synthesis. |
| Nucleotide Metabolism / Synthesis | PWY-7228 | control | Synthesize Guanosine Nucleotides |
| Nucleotide Metabolism / Degradation | P164-PWY | Case | It degrades purine bases stepwise under anaerobic conditions, ultimately producing products such as uric acid, thereby enabling the recycling of purine nucleotides. |
| Carbohydrate Metabolism / Synthesis | GLYCOGENSYNTH-PWY | Case | It synthesizes glycogen using ADP-D-glucose, storing glucose in the form of glycogen to release glucose for energy supply when the organism needs it. |
|  | OANTIGEN-PWY | Case | In Escherichia coli (E. coli), it synthesizes the building blocks for O-antigens. O-antigens are components of lipopolysaccharides (LPS) in Gram-negative bacteria and are associated with properties such as bacterial antigenicity and immunogenicity. |
|  | UDPNAGSYN-PWY | Case | It synthesizes UDP-N-acetyl-D-glucosamine, which is an important precursor for the synthesis of biological macromolecules such as bacterial cell wall peptidoglycans and glycoproteins. |
| Carbohydrate Metabolism / Degradation | PWY-7456 | Case | It decomposes mannan into small-molecule carbohydrates that can be utilized by cells, providing carbon sources and energy for cells. |
|  | RHAMCAT-PWY | control | It degrades L-rhamnose stepwise, producing intermediate products that can enter cellular metabolic pathways and providing an energy and material basis for cellular metabolism. |
| Lipid Metabolism / Synthesis | PWY-5667 | control | It synthesizes CDP-diacylglycerol, which is a crucial intermediate for the synthesis of phospholipids such as phosphatidylinositol and is involved in processes like cell membrane construction and cellular signal transduction. |
|  | PWY-6703 | control | It synthesizes preQ0, a key intermediate in the biosynthetic process of queuosine. Queuosine is a special modified nucleoside present in tRNA, and it plays a crucial role in maintaining the structural stability of tRNA and enabling its functional performance. |
|  | PWY-922 | control | It synthesizes mevalonic acid, which is a key intermediate in the synthesis of various biologically active substances such as cholesterol and terpenes. |
|  | PWY0-1319 | control | It is similar to CDP-diacylglycerol Biosynthesis I, but may differ in aspects such as the substrates, enzymes, or regulatory mechanisms involved in synthesis, and provides raw materials for phospholipid synthesis. |
| Aromatic Hydrocarbon Metabolism / Degradation | PWY-5180 | control | It converts toluene into intermediate products such as o-cresol, then degrades them stepwise into inorganic substances like carbon dioxide and water, thereby achieving the harmless treatment of toluene. |
|  | PWY-5182 | control | It degrades toluene via intermediate products such as 4-methylcatechol using different enzyme systems. |
| Energy Metabolism | PWY-6969 | Case | It uses 2-oxoglutarate as a key node and interacts with ferredoxin oxidoreductase to drive the tricarboxylic acid cycle, providing energy and intermediate metabolites for cells. |

Table S5 Full Names Corresponding to Pathway Codes

| Pathway Codes | Full Names |
| --- | --- |
| P4-PWY | P4-PWY:_superpathway_of_L-lysine,_L-threonine_and_L-methionine_biosynthesis_I |
| GLYCOGENSYNTH-PWY | GLYCOGENSYNTH-PWY:_glycogen_biosynthesis_I_(from_ADP-D-Glucose) |
| HOMOSER-METSYN-PWY | HOMOSER-METSYN-PWY:_L-methionine_biosynthesis_I |
| METSYN-PWY | METSYN-PWY:_L-homoserine_and_L-methionine_biosynthesis |
| OANTIGEN-PWY | OANTIGEN-PWY:_O-antigen_building_blocks_biosynthesis_(E._coli) |
| P164-PWY | P164-PWY:_purine_nucleobases_degradation_I_(anaerobic) |
| PWY-5104 | PWY-5104:_L-isoleucine_biosynthesis_IV |
| PWY-5180 | PWY-5180:_toluene_degradation_I_(aerobic)_(via_o-cresol) |
| PWY-5182 | PWY-5182:_toluene_degradation_II_(aerobic)_(via_4-methylcatechol) |
| PWY-5347 | PWY-5347:_superpathway_of_L-methionine_biosynthesis_(transsulfuration) |
| PWY-5667 | PWY-5667:_CDP-diacylglycerol_biosynthesis_I |
| PWY-6703 | PWY-6703:_preQ0_biosynthesis |
| PWY-6969 | PWY-6969:_TCA_cycle_V_(2-oxoglutarate:ferredoxin_oxidoreductase) |
| PWY-7228 | PWY-7228:_superpathway_of_guanosine_nucleotides_de_novo_biosynthesis_I |
| PWY-7456 | PWY-7456:_mannan_degradation |
| PWY-922 | PWY-922:_mevalonate_pathway_I |
| PWY0-1319 | PWY0-1319:_CDP-diacylglycerol_biosynthesis_II |
| PWY0-781 | PWY0-781:_aspartate_superpathway |
| RHAMCAT-PWY | RHAMCAT-PWY:_L-rhamnose_degradation_I |
| SER-GLYSYN-PWY | SER-GLYSYN-PWY:_superpathway_of_L-serine_and_glycine_biosynthesis_I |
| UDPNAGSYN-PWY | UDPNAGSYN-PWY:_UDP-N-acetyl-D-glucosamine_biosynthesis_I |

Table S6 Correlation Coefficients between Gut Microbiota and Clinical Indicators, Metabolites and Pathways

| Characteristics | B1 | B2 | B3 | B4 | B5 | B6 | B7 | B8 | B9 | B10 | B11 | B12 | B13 |
| --- | --- | --- | --- | --- | --- | --- | --- | --- | --- | --- | --- | --- | --- |
| WBC | -0.075 | -0.021 | 0.172 | -0.127 | 0.089 | 0.045 | 0.059 | -0.152 | -0.131 | 0.168 | -0.039 | 0.142 | -0.035 |
| RBC | -0.254 | -0.046 | -0.148 | -0.049 | 0 | 0.086 | 0.153 | -0.137 | -0.13 | -0.177 | -0.11 | 0.077 | -0.135 |
| HGB | -0.198 | -0.111 | -0.183 | -0.012 | -0.02 | 0.012 | 0.089 | -0.123 | -0.153 | -0.133 | -0.158 | -0.039 | -0.059 |
| PLT | 0.088 | 0.147 | 0.179 | 0.117 | 0.067 | 0.072 | -0.103 | -0.058 | 0.084 | 0.181 | 0.15 | 0.014 | 0.16 |
| ALT | 0.036 | 0.007 | -0.017 | 0.037 | 0.007 | -0.015 | -0.059 | -0.005 | 0.041 | 0.04 | 0.078 | 0.01 | -0.087 |
| AST | 0.083 | -0.081 | 0.143 | 0.085 | 0.08 | -0.089 | -0.063 | -0.055 | -0.01 | 0.04 | 0.041 | 0.113 | -0.136 |
| GGT | -0.152 | 0.038 | -0.159 | 0.064 | -0.076 | -0.084 | -0.149 | 0.049 | 0.03 | -0.003 | 0.076 | -0.065 | 0.12 |
| ALP | 0.148 | 0.048 | 0.113 | 0.126 | 0.052 | -0.037 | -0.172 | -0.082 | 0.046 | 0.083 | -0.051 | 0.083 | 0.053 |
| TP | -0.02 | 0.033 | 0.172 | 0.034 | 0.173 | -0.037 | 0.063 | -0.145 | -0.075 | 0.134 | 0.145 | 0.069 | -0.18 |
| ALB | -0.094 | -0.012 | -0.164 | -0.048 | -0.043 | -0.136 | 0.038 | -0.164 | -0.026 | -0.057 | 0.19 | 0.06 | -0.07 |
| GLB | 0.017 | 0.026 | 0.276 | 0.082 | 0.205 | 0.032 | 0.061 | -0.07 | -0.035 | 0.168 | 0.069 | 0.014 | -0.137 |
| TBIL | 0.107 | -0.197 | 0.056 | 0.022 | 0.105 | -0.131 | -0.008 | -0.033 | -0.228 | 0.008 | -0.077 | -0.123 | -0.189 |
| DBIL | 0.162 | -0.161 | 0.041 | 0.044 | 0.063 | -0.144 | -0.044 | -0.051 | -0.167 | -0.028 | -0.094 | -0.106 | -0.177 |
| IBIL | 0.05 | -0.201 | 0.063 | -0.009 | 0.119 | -0.103 | 0.032 | -0.015 | -0.244 | 0.043 | -0.066 | -0.12 | -0.187 |
| BUN | -0.147 | -0.081 | -0.078 | -0.052 | -0.033 | 0.052 | 0.096 | 0.031 | -0.103 | -0.12 | -0.193 | -0.04 | -0.093 |
| CRE | -0.114 | -0.069 | -0.078 | -0.015 | 0.2 | -0.128 | 0.12 | -0.156 | -0.128 | -0.105 | 0.073 | -0.038 | -0.005 |
| SUA | -0.186 | -0.02 | -0.116 | 0.003 | 0.044 | -0.132 | 0.056 | -0.009 | -0.116 | 0.037 | -0.04 | -0.042 | 0.034 |
| eGFR | -0.017 | 0.097 | -0.143 | -0.067 | -0.258 | 0.071 | -0.055 | 0.154 | 0.058 | -0.013 | 0.038 | 0.03 | 0.009 |
| TC | -0.054 | 0.058 | 0.072 | -0.011 | -0.046 | -0.108 | 0.099 | 0.058 | 0.062 | 0.18 | 0.006 | -0.06 | 0.044 |
| TG | -0.073 | 0.002 | -0.037 | 0.089 | -0.046 | 0.006 | -0.049 | -0.033 | 0.037 | 0.15 | -0.031 | -0.316 | 0.021 |
| HDL-C | 0.173 | -0.052 | 0.369 | 0.027 | 0.13 | -0.121 | -0.018 | 0.266 | -0.028 | 0.136 | 0.086 | 0.112 | -0.095 |
| LDL-C | -0.111 | 0.169 | -0.1 | -0.073 | -0.11 | 0.027 | 0.026 | 0.124 | 0.153 | 0.211 | 0.079 | -0.051 | 0.068 |
| FBG | -0.038 | -0.142 | -0.077 | 0.038 | -0.142 | -0.223 | -0.025 | 0.106 | -0.028 | 0.226 | -0.036 | -0.118 | -0.055 |
| M1 | -0.07 | -0.06 | 0.062 | 0.09 | 0.12 | -0.061 | 0.025 | -0.029 | -0.188 | 0.01 | 0.067 | -0.178 | -0.049 |
| M2 | -0.121 | -0.055 | -0.047 | 0.119 | 0.014 | -0.005 | -0.104 | -0.15 | -0.015 | 0.066 | 0.03 | -0.231 | -0.142 |
| M3 | -0.095 | -0.113 | -0.052 | 0.216 | 0.058 | -0.039 | -0.006 | -0.092 | -0.083 | -0.009 | -0.147 | -0.358 | -0.192 |
| M4 | 0.002 | -0.106 | 0.063 | 0.061 | 0.095 | -0.085 | 0.038 | -0.125 | -0.12 | 0.068 | -0.031 | -0.115 | -0.197 |
| M5 | -0.091 | -0.01 | -0.181 | 0.082 | -0.229 | 0.009 | -0.028 | -0.094 | 0.222 | -0.06 | -0.006 | -0.148 | -0.014 |
| M6 | -0.053 | -0.152 | 0.114 | 0.196 | 0.179 | -0.069 | 0.002 | -0.114 | -0.044 | 0.065 | -0.113 | -0.164 | -0.061 |
| M7 | 0.105 | -0.03 | 0.107 | 0.118 | -0.013 | -0.12 | 0.105 | -0.036 | 0.067 | 0.032 | -0.03 | 0.027 | 0.065 |
| M8 | -0.034 | -0.073 | -0.09 | -0.069 | -0.084 | 0.042 | 0.041 | -0.075 | 0.009 | -0.192 | -0.116 | -0.045 | -0.044 |
| M9 | 0.134 | 0.186 | 0.102 | -0.012 | 0.042 | -0.02 | 0.048 | 0.036 | -0.019 | 0.077 | 0.103 | 0.101 | -0.03 |
| M10 | 0.076 | 0.047 | -0.053 | -0.082 | 0.006 | 0.155 | -0.107 | 0.027 | 0.18 | -0.067 | 0.019 | -0.074 | 0.098 |
| M11 | 0.085 | 0.095 | 0.009 | 0.032 | -0.118 | 0.157 | -0.025 | -0.133 | 0.123 | 0.055 | 0.196 | 0.132 | -0.116 |
| M12 | 0.248 | -0.01 | 0.135 | 0.203 | 0.008 | -0.044 | -0.104 | 0.091 | 0.105 | -0.06 | -0.131 | 0.033 | -0.041 |
| M13 | -0.066 | -0.066 | -0.094 | -0.109 | -0.146 | 0.14 | -0.085 | -0.043 | 0.159 | -0.139 | 0.042 | -0.026 | 0.169 |
| M14 | 0.072 | -0.004 | 0.025 | -0.043 | -0.052 | -0.001 | 0.042 | 0.015 | -0.062 | -0.015 | 0.199 | 0.089 | -0.046 |
| M15 | 0.158 | 0.041 | -0.017 | -0.003 | -0.026 | -0.081 | 0.075 | 0.011 | 0.07 | -0.138 | 0.136 | 0.118 | -0.01 |
| M16 | -0.048 | 0.151 | -0.023 | -0.142 | -0.089 | 0.02 | 0.099 | 0.068 | -0.058 | 0.227 | 0.127 | 0.112 | 0.037 |
| M17 | -0.067 | -0.067 | -0.037 | 0.019 | 0.005 | -0.034 | -0.011 | -0.077 | -0.149 | -0.03 | 0.084 | -0.112 | -0.015 |
| M18 | 0.025 | 0.031 | -0.085 | -0.032 | -0.05 | 0.11 | -0.153 | 0.104 | 0.288 | 0.016 | 0.033 | -0.002 | 0.026 |
| M19 | -0.205 | -0.119 | -0.091 | -0.124 | -0.11 | 0.047 | 0.092 | -0.089 | -0.035 | -0.064 | 0.034 | -0.02 | -0.045 |
| M20 | 0.079 | 0.013 | 0.048 | 0.001 | 0.072 | -0.15 | -0.02 | -0.055 | -0.034 | -0.07 | 0.168 | 0.256 | -0.228 |
| M21 | -0.113 | 0.108 | -0.099 | -0.113 | -0.118 | -0.076 | 0.165 | 0.096 | -0.01 | -0.067 | -0.047 | 0.057 | -0.015 |
| M22 | -0.032 | 0.102 | -0.092 | -0.263 | -0.174 | 0.005 | 0.057 | 0.011 | -0.03 | 0.095 | -0.195 | -0.055 | 0.154 |
| M23 | -0.119 | 0.029 | 0.134 | 0.032 | 0.228 | -0.015 | 0.033 | -0.014 | -0.159 | -0.02 | 0.111 | 0.1 | -0.141 |
| M24 | -0.06 | 0.02 | 0.015 | 0.086 | 0.039 | -0.034 | 0.041 | 0.109 | 0.113 | 0.005 | 0.099 | 0.081 | -0.131 |
| M25 | 0.122 | -0.009 | -0.09 | 0.082 | 0.019 | 0.166 | 0.048 | -0.108 | 0.027 | -0.038 | 0.049 | -0.04 | -0.22 |
| M26 | 0.118 | -0.121 | -0.04 | -0.045 | 0.072 | -0.207 | 0.048 | -0.001 | -0.073 | -0.062 | 0.029 | 0.055 | -0.071 |
| M27 | -0.07 | 0.005 | -0.115 | -0.075 | -0.055 | 0.146 | 0.128 | -0.119 | 0.012 | -0.038 | 0.113 | -0.017 | -0.118 |
| M28 | -0.104 | 0.092 | 0.006 | 0.113 | 0.02 | 0.024 | -0.098 | 0.01 | 0.044 | 0.076 | 0.036 | -0.063 | 0.053 |
| M29 | -0.058 | 0.032 | 0.036 | 0.141 | 0.057 | -0.075 | -0.07 | -0.026 | -0.008 | 0.146 | 0.076 | 0.035 | 0.032 |
| M30 | 0.051 | 0.085 | -0.059 | -0.102 | -0.005 | -0.091 | -0.068 | -0.034 | -0.061 | 0.034 | 0.16 | 0.057 | -0.077 |
| M31 | -0.169 | 0.013 | -0.081 | -0.206 | -0.189 | 0.012 | 0.027 | 0.169 | -0.017 | 0.036 | -0.136 | -0.014 | 0.131 |
| P1 | -0.143 | -0.016 | -0.038 | -0.216 | -0.075 | 0.021 | 0.146 | -0.04 | -0.181 | -0.101 | -0.003 | 0.122 | -0.017 |
| P2 | -0.071 | 0.029 | -0.11 | -0.032 | -0.161 | -0.086 | 0.042 | 0.192 | 0.147 | 0.019 | -0.024 | 0.122 | 0.046 |
| P3 | -0.089 | -0.116 | 0.011 | -0.15 | 0.038 | 0.018 | 0.19 | -0.013 | -0.11 | -0.034 | 0.033 | 0.152 | -0.067 |
| P4 | -0.075 | -0.124 | 0.003 | -0.115 | 0.032 | 0.02 | 0.169 | -0.037 | -0.087 | -0.021 | 0.009 | 0.102 | -0.081 |
| P5 | -0.19 | -0.087 | -0.15 | -0.163 | -0.076 | -0.027 | 0.21 | 0.025 | -0.128 | -0.149 | 0.003 | 0.055 | 0.014 |
| P6 | 0.074 | 0.12 | -0.002 | 0.018 | -0.075 | -0.057 | 0.013 | 0.031 | 0.273 | 0.034 | -0.063 | 0.055 | -0.002 |
| P7 | -0.125 | 0.03 | -0.138 | -0.193 | -0.124 | 0.082 | 0.055 | 0.025 | 0.146 | -0.043 | -0.057 | 0.167 | 0.122 |
| P8 | -0.181 | -0.102 | -0.114 | -0.081 | 0.027 | 0.083 | 0.095 | 0.01 | -0.063 | 0.078 | -0.06 | 0.187 | -0.101 |
| P9 | -0.181 | -0.102 | -0.114 | -0.081 | 0.027 | 0.083 | 0.095 | 0.01 | -0.063 | 0.078 | -0.06 | 0.187 | -0.101 |
| P10 | -0.096 | -0.148 | -0.01 | -0.123 | 0.049 | 0 | 0.173 | -0.043 | -0.11 | -0.037 | 0.014 | 0.089 | -0.078 |
| P11 | -0.101 | -0.215 | 0.075 | 0.04 | 0.075 | -0.173 | 0.21 | -0.188 | -0.127 | 0.017 | -0.024 | 0.051 | -0.104 |
| P12 | -0.074 | -0.127 | 0.064 | 0.008 | 0.135 | -0.077 | 0.192 | -0.194 | -0.145 | -0.003 | 0.006 | -0.031 | -0.033 |
| P13 | -0.081 | -0.01 | 0.022 | 0.002 | -0.028 | -0.052 | 0.113 | 0 | 0.048 | 0.011 | 0.005 | 0.064 | -0.009 |
| P14 | -0.107 | -0.268 | 0.076 | 0.048 | 0.155 | -0.105 | 0.304 | -0.248 | -0.294 | -0.098 | -0.021 | -0.056 | -0.143 |
| P15 | -0.009 | 0.316 | -0.17 | -0.015 | -0.275 | 0.085 | -0.1 | 0.145 | 0.25 | -0.081 | -0.137 | -0.022 | 0.219 |
| P16 | -0.011 | -0.289 | 0.166 | 0.105 | 0.194 | -0.138 | 0.079 | 0.056 | -0.125 | 0.029 | -0.013 | 0.125 | -0.095 |
| P17 | -0.101 | -0.215 | 0.075 | 0.04 | 0.075 | -0.173 | 0.21 | -0.188 | -0.127 | 0.017 | -0.024 | 0.051 | -0.104 |
| P18 | -0.131 | -0.019 | -0.052 | -0.217 | -0.098 | 0.022 | 0.126 | -0.026 | -0.177 | -0.095 | -0.008 | 0.127 | -0.018 |
| P19 | -0.147 | -0.171 | 0.087 | -0.028 | 0.148 | -0.188 | 0.282 | -0.15 | -0.275 | -0.139 | 0.063 | 0.048 | -0.218 |
| P20 | -0.099 | -0.128 | -0.069 | -0.086 | -0.061 | -0.102 | 0.131 | 0.066 | -0.132 | -0.117 | -0.009 | 0.07 | -0.112 |
| P21 | -0.212 | -0.027 | -0.193 | -0.216 | -0.082 | 0.01 | 0.211 | 0.052 | -0.106 | -0.095 | 0.012 | 0.094 | 0.05 |

The red color denote significance levels: P<0.05. B1: Butyricicoccus sp_AM29_23AC, B2: Catenibacterium unclassified, B3: Clostridium fessum, B4: Clostridium unclassified, B5: Clostridium sp_AM22_11AC, B6: Clostridium sp_OM07_10AC, B7: Erysipelatoclostridium ramosum, B8: Faecalibacterium unclassified, B9: Lachnospiraceae bacterium Marseille-Q4251, B10: Mediterraneibacter massiliensis, B11: Ruminococcus sp_AF13_28, B12: Solobacterium unclassified, B13: Staphylococcus unclassified; M1: LysoPC(14:0/0:0), M2: DG(16:1/18:2), M3: DG(17:0/18:2(9Z,12Z)/0:0), M4: DG(18:2/18:2), M5: PC(31:2), M6: DG(20:3(5Z,8Z,11Z)/18:2(9Z,12Z)/0:0), M7: PE(36:4e), M8: PC(34:3p), M9: PC(35:6p), M10: SM(d38:1), M11: ChE(18:3), M12: PC(37:6p), M13: SM(d41:1), M14: PC(38:0e), M15: PI(18:0/20:4(5Z,8Z,11Z,14Z)), M16: PC(39:3), M17: PE(42:2p), M18: CerG1(d18:1/24:1), M19: SM(d44:6), M20: PC(42:7p), M21: TG(16:0/18:1(9Z)/18:1(9Z)), M22: TG(17:0/18:1(9Z)/18:2(9Z,12Z)), M23: TG(18:0e/18:1/18:2), M24: TG(18:1(11Z)/18:2(9Z,12Z)/24:1(15Z)), M25: LPI(20:4), M26: PA(26:5/18:2), M27: PS(18:1(9Z)/20:4(5Z,8Z,11Z,14Z)), M28: PS(18:0/22:4(7Z,10Z,13Z,16Z)), M29: PE(18:0p/22:6), M30: PS(15:0/24:1(15Z)), M31: PE(18:0e/20:4); P1: P4-PWY, P2: GLYCOGENSYNTH-PWY, P3: HOMOSER-METSYN-PWY, P4: METSYN-PWY, P5: OANTIGEN-PWY, P6: P164-PWY, P7: PWY-5104, P8: PWY-5180, P9: PWY-5182, P10: PWY-5347, P11: PWY-5667, P12: PWY-6703, P13: PWY-6969, P14: PWY-7228, P15: PWY-7456, P16: PWY-922, P17: PWY0-1319, P18: PWY0-781, P19: RHAMCAT-PWY, P20: SER-GLYSYN-PWY, P21: UDPNAGSYN-PWY.

Table S7 Evaluation Indicators for the Test Set of the Prediction Model

| Model | Accuracy | Precision | Recall | F1 | Youden | *P* value |
| --- | --- | --- | --- | --- | --- | --- |
| Model 1 | 0.333 | 0.333 | 0.999 | 0.500 | 0.439 | reference |
| Model 2 | 0.778 | 0.615 | 0.889 | 0.727 | 0.460 | 0.008 |
| Model 3 | 0.667 | 0.500 | 0.889 | 0.640 | 0.610 | 0.013 |
| Model 4 | 0.630 | 0.462 | 0.667 | 0.545 | 0.587 | 0.029 |

Model 1: Includes laboratory indicators and general demographic information. Model 2: Builds on Model 1 by adding differential bacteria. Model 3: Builds on Model 1 by adding differential metabolites. Model 4: Builds on Model 1 by adding both differential bacteria and differential metabolites.

Figure S1 Comparison of α diversity

MetS(-/-): the control group; MetS(-/+): the case group.

Figure S2 Comparison of β diversity

MetS(-/-): the control group; MetS(-/+): the case group. PCoA Analysis Based on Distances of Different Samples. a: Bray-Curtis Distance; b: Jensen-Shannon Divergence; c: Pearson-Coefficient Distance; d: Spearman-Coefficient Distance.

Figure S3 Differences in Gut Microbiota Abundance

MetS(-/-): the control group; MetS(-/+): the case group.

Figure S4 Differences in Gut Microbiota Metabolic Pathways

MetS(-/-): the control group; MetS(-/+): the case group.

Figure S5 Volcano Plots for Differential Metabolite Analysis

a: Volcano Plots Under Positive Modes. b: Volcano Plots Under negative Modes. State: Representing the upregulated or downregulated expression levels in the context of MetS.

Figure S6 Z-score Analysis of Differential Metabolite Content

MetS(-/-): the control group; MetS(-/+): the case group. a and b represent the Z-scores of differential metabolites under the positive ion mode and negative ion mode, respectively.

Figure S7 Correlation Heatmap of Gut Microbiota and Clinical Indicators

Gut microbiota and clinical indicators with similar characteristics were clustered. MetS(-/-): the control group; MetS(-/+): the case group. In the grid, the shade of the same color represents the strength of the correlation, with a darker color indicating a stronger correlation. Different colors represent correlations in different directions: red indicates a positive correlation, while blue indicates a negative correlation. Symbols denote significance levels: *: *P*<0.05; **: *P*<0.01; ***: *P*<0.001. WBC: white blood cell; HGB: Hemoglobin; RBC: red blood cell; PLT: platelet count; ALT: glutamic pyruvic transaminase; GGT: gamma-glutamyl transferase; AST: glutamic oxaloacetic transaminase; ALP: alkaline phosphatase; TP: total protein; GLB: globulin; ALB: albumin; TBIL: total bilirubin; IBIL: indirect bilirubin; DBIL: direct bilirubin; BUN: blood urea nitrogen; CRE: creatinine; SUA: serum uric acid; eGFR: estimated glomerular filtration rate; TC: total cholesterol; TG: triglyceride; HDL-C: high-density lipoprotein-cholesterol; LDL-C: low-density lipoprotein-cholesterol; FBG: fasting blood glucose.

Figure S8 Correlation Heatmap of Gut Microbiota and Metabolic Pathways

Gut microbiota and metabolic pathways with similar characteristics were clustered. The remaining annotations are consistent with those in Figure 4.

Figure S9 Correlation Heatmap of Gut Microbiota and Metabolite

Gut microbiota and metabolite with similar characteristics were clustered. The remaining annotations are consistent with those in Figure 4.

Figure S10 Ranking of Feature Importance

MetS(-/-): the control group; MetS(-/+): the case group. HDL-C: high-density lipoprotein-cholesterol; HGB: Hemoglobin; IBIL: indirect bilirubin; FBG: fasting blood glucose; BMI: body mass index; TBIL: total bilirubin; ALP: alkaline phosphatase; ALT: glutamic pyruvic transaminase; DBIL: direct bilirubin; RBC: red blood cell; BUN: blood urea nitrogen; LDL-C: low-density lipoprotein-cholesterol. a: Model 1, includes laboratory indicators and general demographic information. b: Model 2, builds on Model 1 by adding differential bacteria. c: Model 3, builds on Model 1 by adding differential metabolites. d: Model 4, builds on Model 1 by adding both differential bacteria and differential metabolites.

Figure S11 Feature Interpretation of the Prediction Model

MetS(-/-): the control group; MetS(-/+): the case group. HDL-C: high-density lipoprotein-cholesterol; HGB: Hemoglobin; IBIL: indirect bilirubin; FBG: fasting blood glucose; BMI: body mass index; TBIL: total bilirubin; ALP: alkaline phosphatase; ALT: glutamic pyruvic transaminase; DBIL: direct bilirubin; RBC: red blood cell; BUN: blood urea nitrogen; LDL-C: low-density lipoprotein-cholesterol. a: Model 1, includes laboratory indicators and general demographic information. b: Model 2, builds on Model 1 by adding differential bacteria. c: Model 3, builds on Model 1 by adding differential metabolites. d: Model 4, builds on Model 1 by adding both differential bacteria and differential metabolites. SHAP value: positive values indicate a positive impact on the model, increasing the prediction probability; negative values indicate a negative impact on the model, decreasing the prediction probability. Dots: color maps to the magnitude of feature values, and distribution density reflects the contribution pattern of features to the model output.

Figure S12 PCA projection showing group separation based on selected multi-omics features

MetS(-/-): the control group; MetS(-/+): the case group.
